# Repeated stress exposure in mid-adolescence attenuates behavioral, noradrenergic, and epigenetic effects of trauma-like stress in early adult male rats

**DOI:** 10.1038/s41598-020-74481-3

**Published:** 2020-10-21

**Authors:** Lauren E. Chaby, Nareen Sadik, Nicole A. Burson, Scott Lloyd, Kelly O’Donnel, Jesse Winters, Alana C. Conti, Israel Liberzon, Shane A. Perrine

**Affiliations:** 1 Department of Psychiatry and Behavioral Neurosciences, Wayne State University School of Medicine, Detroit, MI USA; 2 Department of Psychiatry, University of Michigan, Ann Arbor, MI USA; 3 Research Service, John D. Dingell VA Medical Center, Detroit, MI USA; 4 Department of Psychology, University of Colorado, Colorado Springs, CO USA; 5 Department of Neurosurgery, Wayne State University School of Medicine, Detroit, MI USA; 6 Department of Psychiatry, Texas A&M College of Medicine, Bryan, TX USA

**Keywords:** Cognitive neuroscience, Epigenetics in the nervous system, Learning and memory

## Abstract

Stress in adolescence can regulate vulnerability to traumatic stress in adulthood through region-specific epigenetic activity and catecholamine levels. We hypothesized that stress in adolescence would increase adult trauma vulnerability by impairing extinction-retention, a deficit in PTSD, by (1) altering class IIa histone deacetylases (HDACs), which integrate effects of stress on gene expression, and (2) enhancing norepinephrine in brain regions regulating cognitive effects of trauma. We investigated the effects of adolescent-stress on adult vulnerability to severe stress using the single-prolonged stress (SPS) model in male rats. Rats were exposed to either (1) adolescent-stress (33–35 postnatal days) then SPS (58–60 postnatal days; n = 14), or (2) no adolescent-stress and SPS (58–60 postnatal days; n = 14), or (3) unstressed conditions (n = 8). We then measured extinction-retention, norepinephrine, HDAC4, and HDAC5. As expected, SPS exposure induced an extinction–retention deficit. Adolescent-stress prior to SPS eliminated this deficit, suggesting adolescent-stress conferred resiliency to adult severe stress. Adolescent-stress also conferred region-specific resilience to norepinephrine changes. HDAC4 and HDAC5 were down-regulated following SPS, and these changes were also modulated by adolescent-stress. Regulation of HDAC levels was consistent with the pattern of cognitive effects of SPS; only animals exposed to SPS without adolescent-stress exhibited reduced HDAC4 and HDAC5 in the prelimbic cortex, hippocampus, and striatum. Thus, HDAC regulation caused by severe stress in adulthood interacts with stress history such that seemingly conflicting reports describing effects of adolescent stress on adult PTSD vulnerability may stem in part from dynamic HDAC changes following trauma that are shaped by adolescent stress history.

## Introduction

Stress during development is so prevalent that it in some populations the majority of children experience at least one traumatic event^1–3^. Stressful events have diffuse effects on the adolescent transition into adulthood, including impairments in health, financial and academic success, and risk of drug taking^4,5^. In clinical populations, decades of research have used a cumulative load model which conceptualizes effects of stress across the lifespan as conferring accruing risk for pathological stress responses^6,7^. Clinical studies have shown adolescent-stress can have lasting and delayed effects, which interact with environmental factors to promote vulnerability or resilience to psychopathology^1,8–12^, reviewed in^13^.

Clinical observations regarding the contribution of adolescent-stress to adult vulnerability to PTSD are by nature correlative, with inherent challenges in defining the timing of stress exposure in retrospect. Additionally, accounting for the specific timing of developmental stress is challenging given variability in stress duration, chronicity, predictability, and the non-linearity of maturational processes within adolescence, resulting from puberty and region-specific brain maturation that is both progressive and regressive^14,15^. These limitations have made the effects of adolescent-stress difficult to define. In parallel, preclinical evidence has demonstrated that adolescent-stress can enhance^16–19^, attenuate^16,20^, or have no detectable effect on cognitive and endocrine features of adult stress responsivity^17–19^; reviewed in^21,22^. One key mechanism by which adolescent-stress may enhance vulnerability to psychopathology is epigenetic modification, including the removal of acetyl groups by histone deacetylases (HDACs)^23^. HDACs reduce transcriptional access and decrease gene expression, thereby acting as epigenetic repressors which modulate behavior and memory^13,24^. HDACs are grouped into classes based on homology, and function independently or cooperatively to achieve rapid, complex changes in gene regulation^25^. HDAC regulation of gene transcription is necessary for adolescent development but can be regulated by stress exposure^26,27^, for example, HDAC4 and HDAC5 are class IIa HDACs which are environmentally regulated and modulate stress responsivity^28,29^. Early stress can lastingly regulate expression of HDACs 1,3,7,8^24,30^, yet, the effects of adolescent-stress on adulthood levels of HDACs are unknown.

Among HDACs, HDAC4/5 are particularly relevant to our inquiry because (1) several studies have linked these targets to effects of environmental context on complex cognition and (2) dysregulation of HDAC4 and HDAC5 can contribute to the development of psychopathology^28,29,31–34^. For example, HDAC4 is linked to heightened fear expression during fear extinction learning through an interaction with PTSD status^33^. HDAC5 has been proposed as a central integrator of the effects of adverse environmental stimuli on chromatin and gene expression^31^. Further, an HDAC5 target, NPAS4, appears to regulate neural activity following stress and contextual memory formation^29,35,36^. Broad pharmacological inhibition of class I and II HDACs can enhance fear memory reconsolidation or fear extinction, including extinction learning, following exposure to the preclinical model of severe stress called single-prolonged stress (SPS)^37–40^. Deficits in the ability to recall and apply extinction learning to attenuate fear responses upon re-exposure to the extinction context, referred to as an extinction retention deficit, is a prominent model of the effects of trauma-like stress that has been characterized in individuals with PTSD and in preclinical models of severe stress^41–45^. Extinction retention deficits in PTSD have been linked to hypoactivation of the medial prefrontal cortex (PFC) and the hippocampus, coupled with amygdala hyperactivation^41,43^.

Another potential key modulator of extinction retention is norepinephrine (NE). NE is also highly relevant to our question of adolescent-stress effects on trauma vulnerability because PTSD is characterized by increased NE in cerebrospinal fluid^46,47^ which is thought to contribute to PTSD symptoms including aberrant fear memory and extinction learning^48^, hyperarousal, re-experiencing, and sleep disturbance^49,50^. Additionally, NE regulation develops throughout adolescence, such that the developmental timing of stress exposure can shape NE systems as well as the effects of NE on cognition, extinction learning^54–56^, and potentially dysregulated memory in PTSD^51–54,57,58^. Further, NE regulation is dynamic and can change in magnitude and direction with compounding stress^59^. SPS affects the noradrenergic system^50^; by increasing NE in the prefrontal cortex, amygdala^60^, and hippocampus^61^. There are also region-specific patterns of NE change following developmental stress^23,52,62^. Thus, we aimed to determine the effects of adolescent-stress followed by adult trauma-like stress on NE in the prelimbic cortex (PLC), hippocampus, striatum, and amygdala in the context of extinction retention.

Here, we investigate the effects of adolescent-stress on cognitive, noradrenergic, and epigenetic effects of trauma-like stress exposure in adulthood using a preclinical model of a deficit found in PTSD. Specifically, we tested the effects of adolescent-stress and adult severe stress (SPS) on extinction retention and region-specific levels of HDAC4, HDAC5, NPAS4, and NE. We predicted that adolescent-stress exposure would increase vulnerability to adverse effects of severe stress, such that animals exposed to adolescent-stress prior to SPS would show impaired extinction retention. We also predicted that the extinction retention deficit would co-occur with (1) lasting decreases in HDAC4/5 and (2) elevated NE. We further predicted that molecular changes, in concert with prominent hypotheses of the cumulative effects, would be greatest following exposure to both adolescent-stress and SPS in adulthood.

## Methods

### Subjects and housing

**Figure 1:**
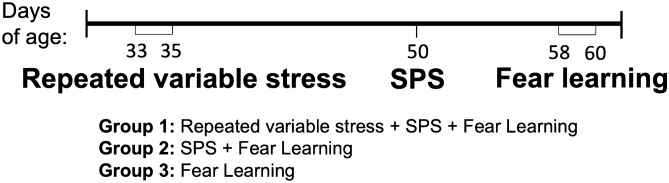
Timeline of procedures. Repeated variable stress consisted of repeated, unpredictable exposure to visual, olfactory, and auditory predation cues (a swooping hawk model, fox urine, large cat vocalizations). The single prolonged stress (SPS) model is defined by three stressors in succession (restraint, forced swim, ether exposure). Following SPS, rats were socially isolated for seven days. During fear learning testing, rats were first trained to associate a tone with a shock, then the tone was repeatedly presented in a novel context to facilitate extinction learning. Rats were then returned to the second context to test the retention of extinction learning. The day following extinction retention testing, brains were collected for region-specific measurement of HDAC4, HDAC5, NE, and NPAS4. All groups were age-matched for SPS, fear learning, and time in the laboratory; groups 2 and 3 were reared in the laboratory under control conditions.

Male Sprague–Dawley CD rats (n = 36) were obtained at 30 days of age from Charles River Laboratories (Kingston, NY). Upon arrival, rats were pair-housed and assigned to one of three groups: (1) adolescent-stress and SPS in early adulthood (n = 14), (2) control rearing and SPS in early adulthood (n = 14; i.e. SPS only), or (3) no stress exposure (n = 8). A timeline of procedures is depicted in Fig. 1. Following arrival, rats were given 5 days to acclimate before handling (20–22 °C; 50% relative humidity; 12:12 light/dark cycle; lights on at 0600 h). Animals were housed in standard plastic cages (20 cm × 26 cm × 45 cm) with wood chip bedding; cleaned weekly, except following SPS. Standard rat chow (LabDiet 5001, 23% protein) and tap water were available ad libitum. To control for circadian rhythms, testing started a minimum of 3 h after the beginning of the light cycle and completed within 4 h of the start of the test. Testing order was pseudo-randomized (randomized within blocks), and treatment groups were evenly distributed during the first and last hours of the testing. To minimize disturbance, the experimenter was not in the room during fear conditioning procedures or adolescent stressor administration. Procedures were approved by the VA Ann Arbor Healthcare System Institutional Animal Care and Use Committee (#1312-004); all procedures were performed in accordance with the relevant guidelines and regulations.

### Repeated variable stress in adolescence

Rats were exposed to repeated variable stress in mid-adolescence, from 33 to 35 postnatal days of age. The repeated variable stress paradigm encompassed three stressors, each representing a different modality of predation cues (visual: a swooping hawk model, olfactory: fox urine, auditory: large cat vocalizations, stressors described in Table 1). Rats encountered 1 stressor per day, for 3 consecutive days. Stressor timing was varied (i.e. unpredictable) and counterbalanced such that half of the rats from each age group experienced 2 stressors during the light phase and 1 during the dark phase with the other half experiencing 1 stressor during the light phase and 2 during the dark phase.

**Table 1:** Adolescent repeated variable stressor descriptions and timeline.

Day of age at exposure	Repeated variable stressor	Duration (min)
33	Rats were exposed to an open arena (63 cm × 93 cm × 93 cm). After a 1 min delay, a model falcon (122 cm wingspan) was moved over the arena in a pendulum motion for 2 min, then moved to a “perching” position approx 1.7 m above the rats. After an additional 7 min rats were removed. Groups of 6 (Chen et al.^65^)	15
34	Tink’s Red Fox-P was sprayed onto cotton balls and placed in plastic mesh containers in the home cage (Fendt and Endres^66^)	60
35	Vocalizations of bobcats (*Lynx rufus*), mountain lions (*Puma concolor*), domestic cats (*Felis catus*), lions (*Panthera leo*), and tigers (*Panthera tigris*) were played to rats in their home cage (Chaby et al.^19^)	60

### Single prolonged stress

SPS has been used for two decades to model PTSD traits^63,64^; reviewed in^50^. In SPS, rats are exposed to three stressors in succession followed by isolation for 7-days [additional detail in supplementary methods and^44,64,65^]. Briefly, rats were restrained for 2 h, then forced to swim (23–24 °C) in a 68 × 56 × 45 cm opaque plastic container in groups of 6–8 for 20 min. Rats were then towel-dried and given 15 min to recuperate with a heat source. Next, rats were exposed to diethyl ether vapors in a desiccator until loss of consciousness. Finally, rats were individually-housed in clean cages and undisturbed for 7-days^44,67^. SPS-induced neuroendocrine effects, including HPA negative feedback and glucocorticoid receptor mRNA expression, are only evident a 7-day quiescent period of SPS^63,67^. Isolation can have neuroendocrine effects, thus control rats were isolated to account for potential housing effects which could otherwise confound group comparisons^68^.

### Fear learning, extinction, and extinction retention testing

SPS can induce an extinction retention deficit, in which fear is enhanced after a fear association has been extinguished, indicating the dominance of fear memory over extinction memory^44,65,67^. To test extinction retention, rats were trained on the 8th day following SPS to associate a tone with a shock across five shock-tone pairings in a fear conditioning chamber (day 1: fear conditioning; shock features: 1 s, 1 mA; tone: 10 s, 1 kHz, 80 dB, 60 s inter-trial-interval^65,69^). Freezing responses were quantified as a proxy of fear^44,70^. On the 9th day after SPS, rats were presented with the same tone 30 times without the shock to extinguish fear responses in a second context (day 2: fear extinction). The second context was differentiated by novel auditory, visual, tactile, and olfactory cues^65,69^. Finally, rats were returned to the extinction context on the 10th day after SPS and were re-exposed to the tone without the shock for 10 presentations (day 3: extinction retention). Additional details are provided in the supplementary methods, based on^65^. Freezing was defined as immobility, > 1 s, with or without small pendulum-like head movements with feet/body/neck immobility without vibrissae movement^69^, as this is indicative of fear in rats^71^ and other small mammals^72,73^.

### Brain dissection for neurochemical analysis

Brains were harvested after rapid decapitation without anesthesia and immediately frozen on dry ice for later processing, on the day following extinction retention testing [methods based on^69^]. To obtain tissue punches of specific regions, brains were thawed at − 20 °C for 10 min. Brains were sliced into 2 mm coronal sections using a chilled stainless-steel rat brain matrix. Bilateral tissue punches (1.5 mm) were obtained from the PLC, dorsolateral striatum, amygdala, and dorsal hippocampus, in accordance with the Paxinos and Watson Rat Brain Atlas, and frozen at − 80 °C for subsequent analysis. Punch hemisphere was randomized at the individual-level for high-pressure liquid chromatography (HPLC) and alternative punches were used for immunoblotting.

### Norepinephrine analysis with high pressure liquid chromatography (HPLC)

Whole tissue NE levels were measured with HPLC [methods based on^69,74^]. Tissue punches were suspended in 50 μL of 0.2 N HClO4, then sonically disrupted and centrifuged (4 °C; 12,300 rpm; 10 min). Next, 25 μL aliquots of supernatant were loaded into a Dionex Ultimate 3000 HPLC system for analysis (Thermoscientific, Waltham, MA). Thermoscientific TEST Mobile Phase flowed in the column at a rate of 0.6 mL/min; containing acetonitrile, phosphate buffer, and an ion-pairing reagent. Coulometric electrochemical detection was achieved with a dual electrode cell set at − 175 mV (reference) and 300 mV (working). Chromatograms were analyzed using Dionex Chromeleon software (version 7); a detection threshold was set at 3 times the average height of 4 solvent peaks (neurochemicals below this threshold were omitted from further analysis). Absolute values of NE were determined by comparison with external standard (Sigma-Aldrich, St. Louis, MO) run in parallel and in duplicate at the beginning and the end of each run. NE levels were corrected using frozen tissue weight to obtain total concentration, expressed as ng neurochemical/mg tissue weight.

### Immunoblotting for HDAC4, HDAC5, and NPAS4 protein levels

Tissue punches were homogenized in lysis buffer (20 mM Tris [pH 7.5], 150 mM NaCl, 2 mM EDTA, 1% Triton X-100, 10% glycerol with protease and phosphatase inhibitors) then centrifuged (4 °C; 10,000 rpm; 10 min). Total protein content of each sample was determined with a Pierce 660 nm Protein Assay (ThermoFisher). Standardized amounts of protein (5 µg) were loaded into 4–12% sodium dodecyl sulfate–polyacrylamide gels and proteins were separated by electrophoresis. Separated proteins were transferred to nitrocellulose membrane by region using a semi-dry transfer apparatus (15V; 40 min; Invitrogen, NY). Nitrocellulose membranes were exposed to antibodies against HDAC4 (rabbit monoclonal, Cell Signaling Technology, #7628; 1:3000^75^) followed by HDAC5 (rabbit polyclonal, Proteintech, 16166-1-AP; 1:1000^76^), then β-Actin (mouse monoclonal, LI-COR, 926-42212; 1:3000) and NPAS4 (goat polyclonal, Abcam, #ab109984; 1:2000) in Odyssey blocking buffer in Tris-buffered saline containing 0.1% sodium azide (LI-COR). Secondary antibodies were used for visualization (HDACs: goat polyclonal anti-rabbit, LI-COR, 1:2000; Actin: goat polyclonal anti-mouse, LI-COR, 1:3000^77^) then blots were scanned on an Odyssey CLx Near-Infrared Fluorescence Imaging System (LI-COR); reference blots in supplementary Figure S1. Following visualization of each secondary antibody, band intensity was quantified with Image J software (NIH) using densiometric analysis for each band, minus the blot background signal for each band^78^. Total protein signals were normalized to β-actin signals and are presented as a ratio to β-actin.

### Data analysis

Percent freezing during each fear learning testing phase was analyzed with a separate repeated measure analysis of variance (RMANOVA), with stress condition and time as fixed effects. Fear extinction (30 trials) and extinction retention (10 trials), due to their length, were separated into an early phase (first half of trials) and late phase (second half of trials)^44,69,79^. If a main effect was detected, groupwise univariate ANOVAs were used to compare each group directly. One rat from the SPS-only group was omitted from behavioral analysis because the video system malfunctioned and did not provide a recording for that animal. To evaluate NE, HDAC4/5, and NPAS4, univariate general linear models were used with adolescent-stress and SPS as fixed effects. If analytes were below the threshold for detection in HPLC the individual measurement was omitted from analysis, these included: one SPS-only animal for the hippocampus; one control and one SPS-only animal in the prelimbic cortex; three control and two rats from each SPS group in the striatum. For the NPAS4 PLC nitrocellulose membrane, two samples were obscured by hyper-staining and were omitted: one SPS-only and one adolescent-stress animal. Analyses were run using IBM SPSS Statistics v.24; values are reported as means ± standard error.

## Results

### Fear learning

**Figure 2:**
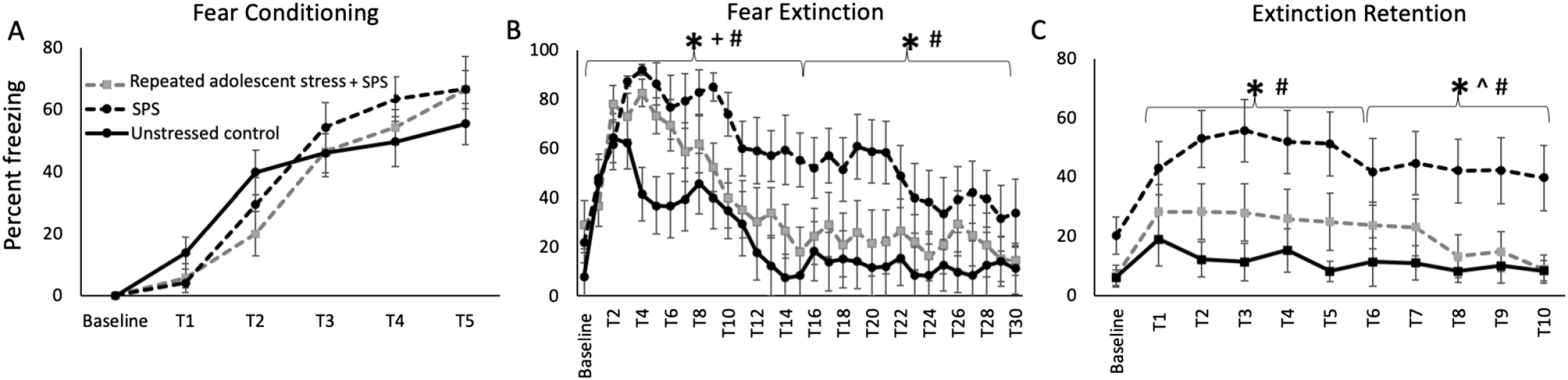
Effects of repeated variable stress during mid-adolescence on fear cognition after traumatic-stress in adulthood. (**A**) All rats showed equivalent fear learning regardless of stress history. (**B**) Fear behavior during fear extinction learning was heightened in rats exposed to SPS stress in adulthood compared with control rats (^#^*P* < 0.05). Exposure to the combination of adolescent-stress and adult SPS increased freezing in the early phase of fear extinction learning, compared with control rats (^+^*P* < 0.05), but this effect abated over time and was not present during the second half of extinction learning. (**C**) Exposure to adult SPS without prior adolescent-stress enhanced fear behavior during both extinction retention test phases (^#^*P* < 0.05). Conversely, the combination of repeated variable stress in adolescence and adult SPS enhanced the retention of safety information during the late phase of extinction retention compared with traumatic stress alone (^^^*P* < 0.05).

All groups showed equivalent increases in fear during the fear conditioning phase (treatment: *F*_1,29_ = 0.29, *P* = 0.75, time x treatment: *F*_1,29_ = 2.94, *P* = 0.07, time: *F*_1,29_ = 140.84, *P* < 0.01; Fig. 2A).

During fear extinction there was a main effect of treatment during the first phase (*F*_1,29_ = 5.95, *P* < 0.01, time x treatment: *F*_1,29_ = 1.99, *P* < 0.01, time: *F*_1,29_ = 14.24, *P* < 0.01) and the second phase (*F*_1,29_ = 3.42, *P* < 0.05, time x treatment: *F*_1,29_ = 1.21, *P* = 0.21, time: *F*_1,29_ = 2.56, *P* < 0.01; Fig. 2B). Group-level comparisons within extinction phase demonstrated that SPS exposure increased fear responses during extinction compared with control rats (no stress) in the first phase (SPS vs. control: *F*_1,19_ = 11.31, *P* < 0.01) and the second phase (SPS vs. control: *F*_1,19_ = 5.74, *P* < 0.03). SPS also decreased the rate of extinction learning compared to controls in the first phase (time × treatment: *F*_1,19_ = 2.88, *P* < 0.01), but this attenuated to a trend in the second phase (*F*_1,19_ = 2.88, *P* = 0.096). Exposure to adolescent-stress (AS), compared to SPS alone, prevented the development of deficits in extinction learning in the first phase (SPS vs. AS + SPS: *F*_1,22_ = 4.07, *P* = 0.05; time × treatment: *F*_1,22_ = 1.83, *P* = 0.03), at the level of a trend in the second phase (SPS vs. AS + SPS: *F*_1,22_ = 3.16, *P* = 0.089, no interaction detected). In both phases animals exposed to AS and SPS did not differ from controls (phase 1: AS + SPS vs. control: *F*_1,18_ = 2.26, *P* = 0.15, interaction: *F*_1,18_ = 1.24, *P* = 0.25; phase 2: AS + SPS vs. control: *F*_1,18_ = 0.66, *P* = 0.43, interaction: *F*_1,18_ = 0.56, *P* = 0.89).

In the first half of the extinction retention testing there was a main effect across the three treatments (*F*_1,29_ = 3.32, *P* = 0.05, interaction and time effects *P* > 0.05; Fig. 2C). Subsequent group comparisons showed that SPS enhanced freezing during the first half of extinction retention compared with unstressed rats (SPS vs. control: *F*_1,16_ = 6.94, *P* = 0.02, interaction: *F*_1,16_ = 2.42, *P* < 0.06), but rats exposed to AS and SPS did not differ from either the SPS only or unstressed groups (*P* > 0.05). For the second phase of extinction retention testing there was a main effect of treatment (*F*_1,29_ = 3.70, *P* = 0.04, no interaction or time effects, *P* > 0.05). SPS rats continued to show elevated freezing responses compared with unstressed rats (SPS vs. control: *F*_1,16_ = 5.46, *P* = 0.03, no interaction detected). However, the combination of AS and SPS decreased freezing compared with SPS alone, i.e. increased extinction retention (SPS vs. AS + SPS: *F*_1,22_ = 4.09, *P* = 0.05, no interaction detected). Further, the extinction retention freezing scores of rats exposed to AS prior to SPS did not differ from unstressed rats (AS + SPS vs. control: *F*_1,20_ = 0.50, *P* = 0.49, no interaction detected).

### Norepinephrine levels

**Figure 3:**
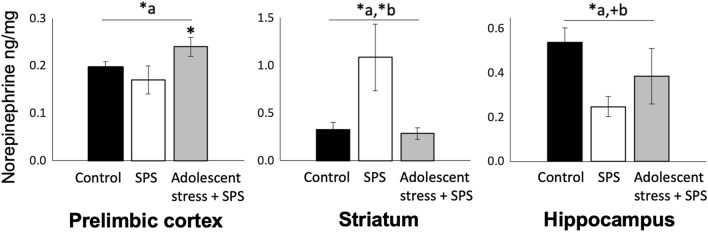
Norepinephrine levels in brain regions mediating fear cognition in adult male rats that were exposed to either repeated variable stress in adolescence followed by SPS, only age-matched SPS exposure, or unstressed control conditions. (**a**) indicates an effect of adolescent-stress exposure; (**b**) indicates an effect of SPS exposure in adulthood; (*P < 0.05, ^+^*P* = 0.11). In the prelimbic cortex of young adult rats, following extinction retention testing, norepinephrine levels were elevated by the combination of repeated variable stress and adult SPS but were not affected by adult SPS alone. In the striatum, adult SPS elevated norepinephrine, but this effect was reversed by prior exposure to repeated variable stress in mid-adolescence. In the hippocampus, exposure to adult SPS decreased levels of norepinephrine; there was a trend towards a norepinephrine increase from adolescent-stress exposure.

In the PLC, SPS exposure alone did not affect NE levels (SPS main effect: F_1,29_ = 2.46, *P* = 0.13). However, PLC NE was elevated by the combination of AS and SPS (AS main effect: F_1,29_ = 4.78, *P* = 0.04, Fig. 3). In the hippocampus, SPS exposure decreased NE (SPS main effect: *F*_*1,28*_ = 8.21, *P* = 0.01). Conversely, exposure to the combination of AS and SPS did not affect hippocampal NE (AS main effect: *F*_*1,28*_ = 2.64, *P* = 0.11). In the striatum, exposure to SPS elevated NE (SPS main effect: *F*_*1,26*_ = 4.42, *P* < 0.05), but this effect was reversed by AS (AS main effect: *F*_*1,29*_ = 7.07, *P* = 0.01). In the amygdala, seven samples were below thresholds for detection, such that NE could not be accurately determined for this region.

### HDAC4, HDAC5, and NPAS4 protein levels

**Figure 4:**
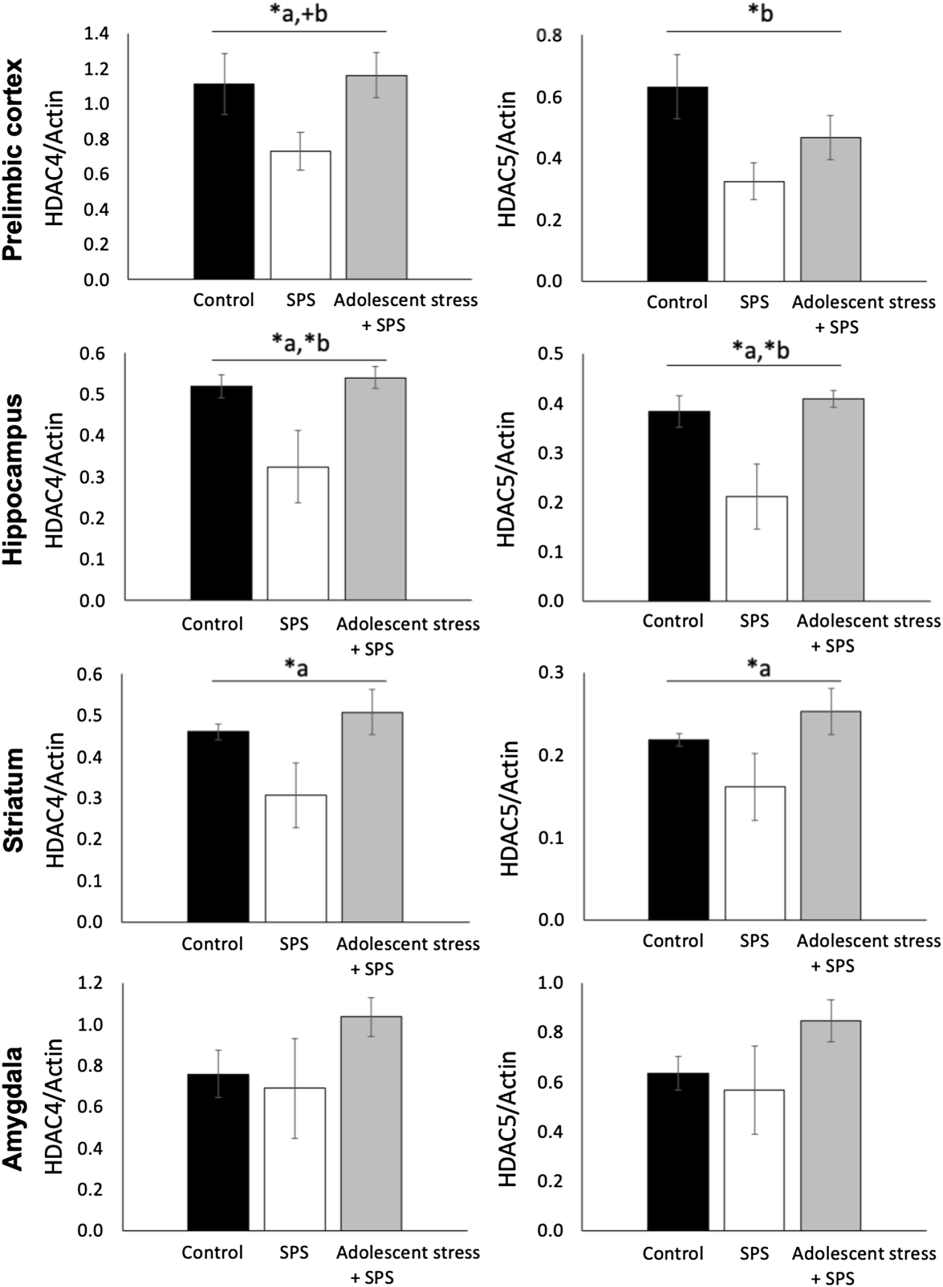
Histone deacetylase (HDAC) 4 and 5 levels in brain regions mediating fear cognition in adult male rats that were exposed to either repeated variable stress in adolescence followed by SPS in adulthood, only age-matched SPS exposure, or unstressed control conditions. (**a**) indicates an effect of adolescent-stress exposure; (**b**) indicates an effect of adult SPS exposure; (*P < 0.05, ^+^*P* = 0.10). In the prelimbic cortex, SPS exposure decreased levels of HDAC4 and HDAC5, while prior exposure to repeated adolescent-stress mitigated these effects (HDAC4 levels were shaped by exposure to adolescent-stress; HDAC5 levels were decreased by SPS). In the hippocampus, HDAC4 and HDAC5 levels were decreased by SPS, but this effect was reversed by prior adolescent-stress. In the striatum, exposure to repeated variable stress in mid-adolescence followed by SPS increased HDAC4 and HDAC5, whereas SPS alone decreased HDAC4 and HDAC5; however, only adolescent-stress had a statistically detectable effect. In the amygdala, neither mid-adolescent stress nor SPS had detectable effects on HDAC levels (*P* > 0.15).

In the PLC, HDAC4 levels were affected by exposure to AS (AS main effect: *F*_*1,23*_ = 4.00, *P* = 0.05) whereas SPS had a trend-level effect (SPS main effect: *F*_*1,23*_ = 2.92, *P* = 0.10, Fig. 4). Compared to controls, SPS decreased PLC HDAC4 levels by 34%, whereas the combination AS + SPS increased PLC HDAC4 levels by 4%. Similarly, compared with the control condition, PLC HDAC5 levels were decreased 49% by SPS and 26% by AS + SPS, but no AS effect was detected (SPS main effect: *F*_*1,23*_ = 6.08, *P* = 0.02; AS main effect: *F*_*1,23*_ = 1.53, *P* = 0.23). The HDAC5 target NPAS4 was not affected by either stress manipulation in the PLC (*P* > 0.51, Supplementary Fig. S1).

In the hippocampus, SPS decreased HDAC4 and HDAC5 levels by 37% and 45%, respectively, compared with unstressed controls (SPS main effect: HDAC4: *F*_*1,25*_ = 5.52, *P* = 0.03; HDAC5: *F*_*1,25*_ = 7.02, *P* = 0.02). Conversely, hippocampal HDAC4 and HDAC5 were increased by 4% and 7% in rats exposed to AS + SPS, respectively, compared to controls (AS main effect: HDAC4: *F*_*1,25*_ = 7.83, *P* = 0.01; HDAC5: *F*_*1,25*_ = 10.73, *P* < 0.01). Hippocampal NPAS4 was not affected by any stress manipulation (*P* > 0.41, Supplementary Fig. S2).

In the striatum, AS shaped HDAC4 and HDAC5 levels (AS main effect: HDAC4: *F*_*1,23*_ = 6.16, *P* = 0.02; HDAC5: *F*_*1,23*_ = 7.74, *P* = 0.01). Compared to controls, AS + SPS increased HDAC4 by 10% and HDAC5 by 16% whereas SPS-alone decreased HDAC4 by 34% and HDAC5 by 26%. However, SPS did not have a statistically detectable effect (*P* > 0.11).

In the amygdala, neither AS nor SPS had detectable effects on HDAC4/5 levels (P > 0.15). Thus, NPAS4 was not evaluated in the amygdala.

## Discussion

We predicted that adolescent-stress exposure would exacerbate extinction retention deficits as well as epigenetic and noradrenergic changes seen in animals exposed to trauma-like stress in adulthood. Contrary to our prediction, adolescent-stress buffered the adverse effects of adult severe stress on extinction retention and HDAC levels (Fig. 2A,B). In line with our predictions, adolescent-stress increased PLC NE following adult SPS, suggesting that adverse adolescent conditions lastingly regulate noradrenergic responses to severe stress in adulthood. In the other regions tested, NE did not conform to our prediction: SPS elevated striatal NE but this effect was not seen if SPS was preceded by adolescent-stress, suggesting buffering effects of adolescent-stress. In the hippocampus, NE was decreased by adult stress exposure, without a detectable effect of adolescent-stress. These results support dynamic, region-specific effects of adolescent-stress on adult noradrenergic signaling rather than additive effects of the combined stress manipulations (Fig. 3). Similarly, adolescent-stress prior to adult-stress had systematic buffering effects on HDAC4 and HDAC5 levels in brain regions that mediate cognitive effects of traumatic stress (Fig. 4). Thus, exposure to stress in adolescence has the capacity to shape the cognitive, noradrenergic, and epigenetic effects of future stress in adulthood.

The effects of adolescent-stress and adult SPS on NE levels were region specific but were not additive in any region tested. In the PLC, the combination of adolescent-stress and adult SPS exposure increased NE, which is consistent with prior evidence that stress exposure increases PLC NE^80,81^, yet SPS alone did not affect PLC NE [similar to^61^]. Thus, regulation of stress-induced PLC NE could be a mechanism by which adolescent-stress precipitates the emergence of adverse cognitive effects of traumatic stress. Our findings extend prior evidence that adolescence may be a period of increased stress sensitivity in NE regulation compared with adulthood^82^, and support that adolescence is a sensitive period for shaping adult responses to stress through region-specific NE regulation. In the hippocampus, SPS decreased NE; hippocampal NE modulates synaptic plasticity and is necessary for the retrieval of contextual memories such that decreased hippocampal NE could inhibit extinction retention^83,84^. In the striatum, adolescent-stress appeared to buffer effects of SPS on NE levels, concurring with the group patterns of extinction retention performance. Post hoc analysis did not reveal individual-level relationships; a design powered for individual-level analysis could elucidate these patterns. The functional significance of increased striatal NE is unclear, but it should be noted that NE changes are dynamic and exposure to shocks, such as those administered during fear conditioning, triggers release of NE from the locus coeruleus thereby increasing NE^58,84^. However, a time-course study has yielded insights into the timing of NE release following shock and has shown a short-term depletion in NE across the brain (including in the PFC and hippocampus), except in the striatum^85^. Overall, changes in NE are dynamic and mediated by pre-existing NE levels and the specifics of task stimuli^86^. To elucidate the relationship between effects of stress history on HDAC4/5 levels and changes in NE demonstrated by the current results, future studies could leverage pharmacological manipulation of NE or HDAC4/5 levels and time-course measurements in limbic and frontal regions and the locus coeruleus. Defining the capacity for stress history to shape effects of trauma on NE is essential given that (1) NE regulation has been implicated in various features of PTSD, including aberrant fear extinction^48^, and (2) therapeutic drugs to either elevate or reduce NE transmission have both had varied success in off-label treatment of PTSD, highlighting key gaps in current knowledge^86^*.* Of interest, administration of a systemic $\upbeta$-noradrenergic receptor antagonist after fear conditioning in male rats can reduce fear behavior during extinction learning and enhance extinction retention, demonstrating a role of NE in extinction learning and memory^87^*.*

Consistent with our predictions and prior clinical evidence of decreased HDAC4 in PTSD^33^, our results demonstrate that SPS decreased HDAC4/5 in brain regions mediating cognitive effects of traumatic stress. Contrary to our prediction, we found that exposure to adolescent-stress prior to adult SPS buffered effects on HDAC4/5. This is in contrast to apparent cumulative effects of adverse childhood experiences on methylation^9–12^, and reflects evidence that while DNA methylation is thought to lead to stable gene repression, certain histone modifications are reversible and regulate dynamic pathways that may present novel therapeutic targets^88^. Our current results suggest that HDAC4/5 expression can be dynamically modulated by both current stress and stress history. Adolescent-stress could buffer SPS-induced changes in HDAC4/5 through three possible mechanisms: (1) preventing changes in HDAC4/5 following SPS, (2) reversing effects of SPS after a temporal delay, or (3) opposing effects that precede SPS exposure (i.e. lasting HDAC4/5 increases). The latter possibility is less congruent with accumulating evidence of dynamic HDAC regulation in response to environmental conditions^31,33^. Adolescent-stress could prevent (1) or reverse (2) effects of SPS on HDAC4/5 by lastingly modulating the sensitivity by which HDAC4/5 are synthesized or degraded. HDAC4 degradation is regulated by the ubiquitin–proteasome system, with stress-dependent activity enabling cells to withstand stress^34,89–91^. The similar patterns of change detected following stress in HDAC4/5 may reflect that both of these can be regulated by the same microRNAs^92,93^. Although we show HDAC4/5 patterns to be similarly affected by stress and stress history, differential HDAC4/5 activity could arise through differential subcellular distribution, actions of histone acetyl transferases, or interaction with downstream targets^94,95^. Here, levels of an HDAC5 target, NPAS4, were not affected by stress manipulation, emphasizing the independent regulation of gene targets and suggesting that NPAS4 may not be a gene target consolidating effects of traumatic stress exposure.

A key extension of the current study would be the inclusion of female rodents. Epigenetic effects of developmental stress can be greater in females than in males^96,97^. Further, HDAC binding and expression in the brain is sex specific, and sex steroids modulate effects of stress on HDAC regulation^33,98–101^. Additionally, humans and rodents show sex specificity in effects of traumatic stress and learned fear responses, including fear conditioning behavior^102–104^. A limitation of the current study is that effects of adolescent-stress are not isolated or directly compared to effects during adulthood or other, earlier developmental stages. Given the extensive maturational changes of systems investigated here, and unknown ontogenetic changes in HDAC4/5, effects of stress during other phases may differ in magnitude or direction. Finally, all neurochemical measures were obtained at a single time-point, such that plasticity is unclear. Time-course studies could determine whether adolescent-stress prevents change in HDAC4/5 following traumatic stress or reverses effects after a delay as well as define effects of stress timing and intensity.

## Conclusion

Our results demonstrate a novel, unanticipated capacity for stress in adolescence to buffer effects of adult trauma-like stress on a cognitive deficit characteristic of PTSD as well as HDAC4/5 and NE levels. Our findings also expand current models of developmental stress with respect to epigenetic regulation in adulthood, by demonstrating that HDAC4 and HDAC5 are dynamically regulated following stress exposure in a manner that reflects stress history. Given this result, and prior evidences that HDAC4/5 have key roles regulating effects of stress on behavior, complex cognition, glucocorticoids, and neural activity, evaluation of HDAC4/5 in the context of resilience and population subgrouping could be informative^29,35,36^. Additionally, our findings suggest that stress history can drive heterogeneity in responses to traumatic stress in adulthood through dynamic HDAC programming effects, which may manifest in clinical inconsistencies in the effects of childhood stress on vulnerability to adverse effects of adult trauma.

## Supplementary information

Supplementary Information

## Data Availability

Data will be made publicly available at the time of publication through ResearchGate: https://www.researchgate.net/profile/Lauren_Chaby/publications.

## Abbreviations

PTSD: Posttraumatic stress disorder
NE: Norepinephrine
HDAC: Histone deacetylase
HDAC4/5: HDAC4 and HDAC5
SPS: Single prolonged stress
