# Supplementary material for: Repeated stress exposure in mid-adolescence attenuates behavioral, noradrenergic, and epigenetic effects of trauma-like stress in early adult male rats
Source: Sci Rep. 2020 Oct 21;10:17935. doi: 10.1038/s41598-020-74481-3 (PMC7578655; doi:10.1038/s41598-020-74481-3)
Supplement: Supplementary file 1 — Supplementary Information [file 41598_2020_74481_MOESM1_ESM.pdf]

Supplementary Materials

Title: Repeated stress exposure in mid-adolescence attenuates behavioral, noradrenergic, and epigenetic effects of trauma-like stress in early adult rats

Authors & affiliations:

Lauren E. Chaby*[a,b], Nareen Sadik[a], Nicole A. Burson[a], Scott Lloyd[a,c], Kelly O'Donnel[d], Jesse Winters[b], Alana C. Conti[c,e], Israel Liberzon[f], Shane A. Perrine[a,c]

[a]Department of Psychiatry and Behavioral Neurosciences
Wayne State University School of Medicine; Detroit, Michigan, United States of America
[b]Department of Psychiatry
University of Michigan; Ann Arbor, Michigan, United States of America
[c]Research Service
John D. Dingell VA Medical Center; Detroit, Michigan, United States of America
[d]Department of Psychology
University of Colorado; Colorado Springs, Colorado, United States of America
[e]Department of Neurosurgery
Wayne State University School of Medicine; Detroit, Michigan, United States of America
[f]Department of Psychiatry
Texas A&M College of Medicine; Bryan, Texas, United States of America

Corresponding author:

*Lauren Chaby
Lauren.Chaby@gmail.com | (617) 203-7356 | 6135 Woodward Ave, IBio, Detroit, MI 48201

Supplementary Methods

Single-prolonged stress (supplementary methods):
Prior to single prolonged stress (SPS), animals were all pair housed for social enrichment. To prevent social buffering following the trauma-like stress exposure, all animals were individually housed following SPS. While SPS animals underwent the SPS procedure, control animals were moved to single housing to account for effects of housing conditions. Animals were not handled or disturbed for feeding/water provisions during the 7-day quiescent period following SPS; rats were provided enough rat chow to last throughout the quiescent period immediately after SPS, had access to water *ad libitum* through a water nozzle at the back of the cage that was fed through piping in the caging rack, and individual rats were isolated in microisolator housing.

Fear learning, extinction, and extinction retention testing (supplementary methods):
Each of the 3 fear learning phases used 8 sound-attenuating boxes, housing individual experimental chambers measuring 30 x 24 x 21cm (MED Associates, St. Albans, VT). All chambers were connected to a computer interface that controlled the experimental contingencies using MedPC software. The floor of each chamber was made of stainless-steel rods measuring 4mm in diameter, spaced 1.5cm apart. The rods were connected to a shock source, which delivered a 1 second 1mA shock as an unconditioned stimulus during the fear learning phase (5 total shocks). All chambers were mounted with a speaker that delivered a 10 second 1kHz 80dB acoustic tone, which was used as the conditioned stimulus. The time interval between each conditioned stimulus (tone) was 1 minute. Each chamber was also equipped with a 15W light, and a fan that provided a 65dB white noise. Trials were video recorded by cameras mounted on the top of each testing chamber and freezing behavior was measured by raters blind to treatment. Freezing was measured continuously, then defined into temporal blocks for each stimulus presentation and intertrial interval (70 second blocks) for analysis. Within each trial block, percent time freezing was calculated as [(time freezing in stimulus block/total time in stimulus block) x 100] (Chaby et al., 2019).

Baseline movement was recorded for 3 minutes during each phase, before animals were presented with tones. For all three phases, animals were transported to the behavioral testing room and allowed to acclimate for 10 minutes in their transport boxes before being transferred to the testing chambers. Transport boxes and testing chambers were cleaned with tap water and dried between animals.

Two contexts were used, differentiated by auditory, visual, tactile and olfactory cues:
Context A (Fear Conditioning): Animals were transferred from their individual home cages to the behavioral testing room, and their individual testing chamber, in lidded black plastic boxes approximately the size of the home cage. Ammonium hydroxide (1%) diluted with tap water acted as a volatile scent cue placed inside of each chamber. A red light was used as a visual cue placed outside of the chambers, oriented towards the chambers. To further differentiate the context, the doors of the sound-attenuating boxes containing the chambers were left open, and the chamber lights and fans were left off.

Context B (Fear Extinction/Extinction Retention): Animals were transferred from their individual home cages to the behavioral testing room, and their individual testing chamber, in lidded white plastic boxes with clean bedding, approximately the size of the home cage. Acetic acid (1%) diluted with tap water acted as a volatile scent cue placed inside of each chamber. Chamber lights were used as a visual cue, and chamber fans acted as an auditory cue. The doors of the sound-attenuating boxes were closed.

Analyses were performed using IBM SPSS Statistics v.24 (SPSS: https://www.ibm.com/products/spss-statistics); figures were generated using SPSS or Microsoft Office (https://www.office.com/).

Supplementary Figures

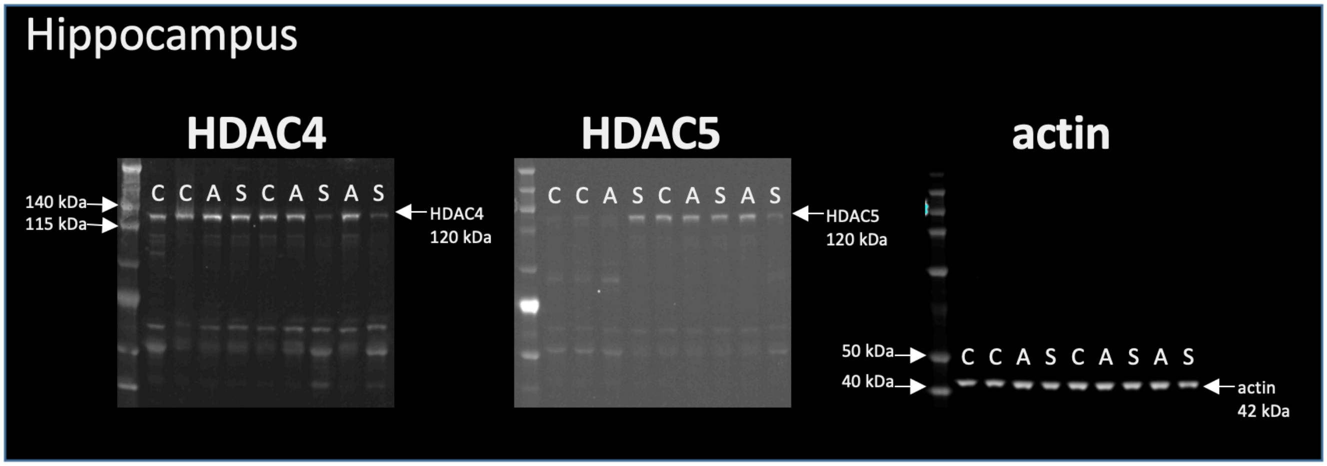

Supplementary Figure 1: Representative images from Western blot analysis of protein levels for HDAC4, HDAC5, NPAS4, and actin (control); depicted here are images of the hippocampus nitrocellulose blots. For the Western blot analysis, tissue punches were homogenized and standardized for protein content. Animal order was randomized to ensure distribution of the treatment groups across the blot (treatment group indicated by C = control animals, S = single prolonged stress exposed animals, A = adolescent stress + single prolonged stress exposed animals). Nitrocellulose membranes were exposed to antibodies against HDAC4 (rabbit monoclonal, Cell Signaling Technology, #7628; 1:3000) followed by HDAC5 (rabbit polyclonal, Proteintech, 16166-1-AP; 1:1000), then b-Actin (mouse monoclonal, LI-COR, 926-42212; 1:3000) and NPAS4 (goat polyclonal, Abcam, #ab109984; 1:2000). Secondary antibodies used for visualization were HDACs: goat polyclonal anti-rabbit, LI-COR, 1:2000; Actin: goat polyclonal anti-mouse, LI-COR, 1:3000. Blots were scanned on an Odyssey CLx Near-Infrared Fluorescence Imaging System.

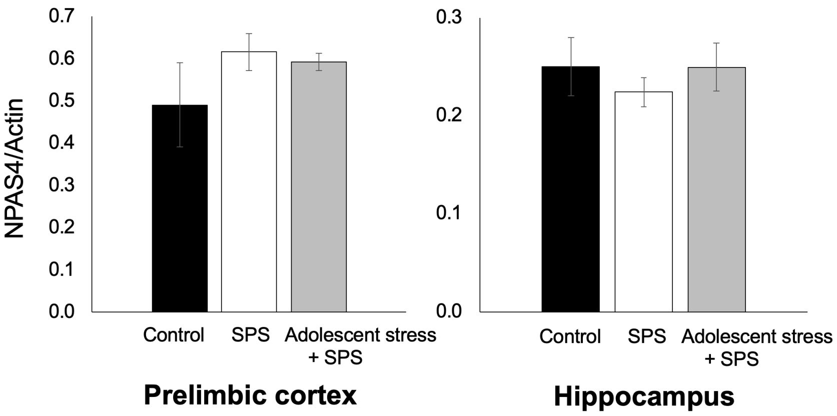

Supplementary Figure 2: NPAS4 levels in adult male rats were not affected following stress in adolescence followed by traumatic-like single-prolonged stress in adulthood (SPS) or SPS exposure alone.
